# Tertiary Lymphoid Structures in Cancer: The Double-Edged Sword Role in Antitumor Immunity and Potential Therapeutic Induction Strategies

**DOI:** 10.3389/fimmu.2021.689270

**Published:** 2021-07-29

**Authors:** Wendi Kang, Zhichao Feng, Jianwei Luo, Zhenhu He, Jun Liu, Jianzhen Wu, Pengfei Rong

**Affiliations:** ^1^Department of Radiology, The Third Xiangya Hospital of Central South University, Changsha, China; ^2^Molecular Imaging Research Center, Central South University, Changsha, China

**Keywords:** tertiary lymphoid structures, tumor immunity, lymphoid neogenesis, bioengineering, immunotherapy, LIGHT

## Abstract

The complex tumor microenvironment (TME) plays a vital role in cancer development and dramatically determines the efficacy of immunotherapy. Tertiary lymphoid structures (TLSs) within the TME are well recognized and consist of T cell-rich areas containing dendritic cells (DCs) and B cell-rich areas containing germinal centers (GCs). Accumulating research has indicated that there is a close association between tumor-associated TLSs and favorable clinical outcomes in most types of cancers, though a minority of studies have reported an association between TLSs and a poor prognosis. Overall, the double-edged sword role of TLSs in the TME and potential mechanisms need to be further investigated, which will provide novel therapeutic perspectives for antitumor immunoregulation. In this review, we focus on discussing the main functions of TLSs in the TME and recent advances in the therapeutic manipulation of TLSs through multiple strategies to enhance local antitumor immunity.

## Introduction

Tumors originate and develop in a complicated and dynamic microenvironment, and there are endothelial cells, immune cells, and stromal cells existing around or within the tumor microenvironment (TME) and interacting with tumor cells (1, 2). Effective antitumor immunity is recognized to require the existence and activation of a variety of immune cells, including B cells, CD8^+^ T cells, and CD4^+^ T cells, etc. This concept is confirmed by the presence of intratumoral tertiary lymphoid structures (TLSs), which are well-organized tumor-infiltrating lymphocyte (TIL) clusters and may generate an advanced immune response (3). As is known to us, immunotherapy can utilize positive feedback to activate the immune system and boost the infiltration of endogenous T cells into tumors and subsequent destruction of tumor cells (4–6). However, only 5%-30% of patients with malignancies exhibit activated intratumoral T cell immunity after anti-programmed cell death protein-1 (PD-1)/programmed death-ligand 1 (PD-L1) immunotherapy (7, 8). This failure is mainly due to the extensive immunosuppressive mechanisms in the TME that lead to the decreased number and dysfunction of infiltrating T cells (9–11).

TLSs are ectopic lymphoid organs that can develop at sites of chronic inflammation, such as those associated with infection and autoimmunity, but also form within the TME (12, 13). TLSs share similar structural and functional characteristics with secondary lymphoid organs (SLOs) (14). However, TLSs lack a capsule and can form in various nonlymphoid tissues, such as stroma and epithelium (15). The prognostic impact of TLSs has been widely explored and most reports have indicated that TLSs are associated with positive immunoreactivity and favorable clinical outcomes in most types of cancers (12, 16–20). For example, TLSs are shown to be associated with relapse-free survival in patients with oral carcinoma or early-stage hepatocellular carcinoma (21, 22). Moreover, germinal centers (GCs) in TLSs may determine the prognostic value of TLSs (23, 24). Even though, TLSs show an association with poor prognosis in a minority of studies (25–27). It is urgent to comprehensively illustrate the function of TLSs in the TME.

SLOs, such as lymph nodes (LNs), provide three-dimensional structures for immune cells to optimize cell-cell interactions and produce an effective immune response (28, 29). Effector T (Teff) cells are activated after being instructed by DCs, and migrate from external draining LNs into the tumor to exert their function (30, 31). Increasing studies have shown that the antitumor immune response originates not only in LNs but also directly in TLSs (32). In general, the cells and molecules that regulate the signaling underlying TLS formation and promote immune responses within TLS remain to be further studied. In this review, we briefly summarize the development of TLSs and focus on discussing the function of TLSs and multiple approaches that had been developed to induce TLS formation.

## Development and Formation of TLSs

**Figure 1:**
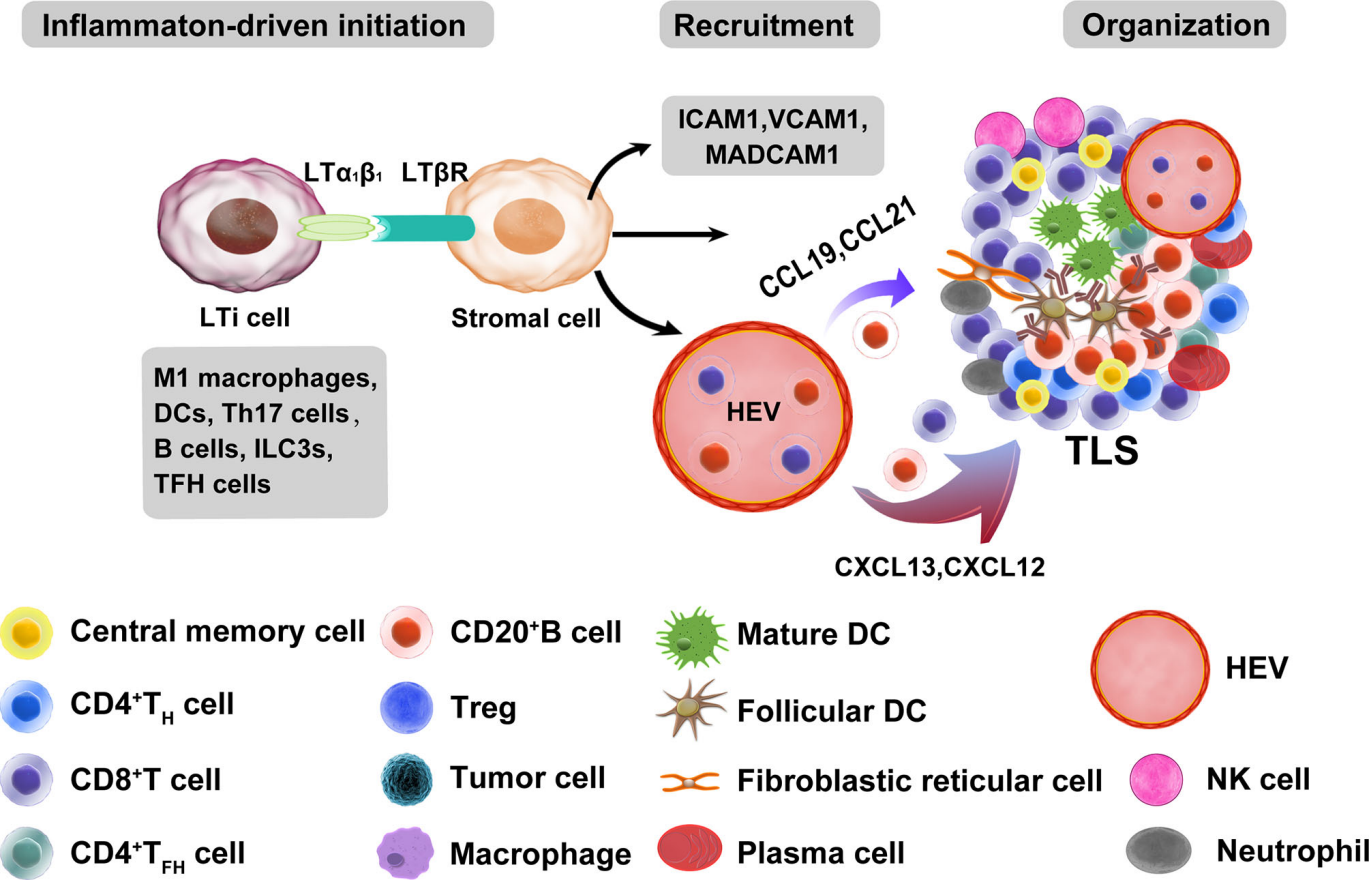
Main molecular and cellular mechanisms of TLS formation in tumors. The development of TLSs is similar to that of SLOs. A chronic inflammatory state is sufficient to induce TLS formation in the absence of lymphoid tissue inducer (LTi) cells. Many immune cells can be used as LTi cells, such as B cells, DCs, M1 macrophages, Th17 cells, ILC3s, and TFH cells. Immune and stromal cell cross-talk mediates TLS formation mainly through the binding of lymphotoxin (LT) αβ and LTβR, which can further release many chemokines (CXCL13, CXCL12, CCL21, and CCL19) and adhesion molecules (VCAM1, ICAM1, and MADCAM1). These chemokines recruit lymphocytes from HEVs and form T and B cell zones. ILC3s, group 3 innate lymphoid cells; VCAM1, vascular cell adhesion molecule 1; ICAM1, intercellular adhesion molecule 1; MADCAM1, mucosal addressable cell adhesion molecule 1.

SLO development is a highly organized process that is initiated and continued during embryogenesis, which has similarities to TLS formation and provides a classical model for understanding TLS development (33). However, there are some differences between the canonical SLOs and TLSs. A chronic inflammatory state is sufficient to induce TLS formation even in the absence of lymphoid tissue inducer (LTi) cells (34), indicating that chronic inflammation may be an important factor that favors lymphoid neogenesis and promotes TLS formation (35). Some studies have revealed that DCs (36), T helper 17 (Th17) cells (37, 38), B cells (39), M1 macrophages (40), and T follicular helper (TFH) cells (41) can initiate TLS neogenesis in various pathological conditions. In addition, group 3 innate lymphoid cells (ILC3s) are associated with ectopic lymphoid aggregates (42). Tumor-infiltrating ILC3s may interact with fibroblasts and lung tumor cells to facilitate cytokine release, contributing to protective TLS formation (43). Lymphotoxin (LT) signaling plays a vital role in TLS formation (44, 45). The LTα1β2-LTβ receptor (LTβR) interaction initiates signaling that leads to the production of various chemokines and adhesion molecules, such as CCL19, CCL21, CXCL13, and CXCL12 (46). CCR7-expressing T cells are recruited by their homologous ligands CCL21 and CCL19, and this recruited population forms a T cell zone. In addition, B cells can express CXCR5 and CXCR4 on their surface to transmigrate to the follicle through the activity of the CXCL12-CXCR4 and CXCL13-CXCR5 axis (47, 48). These findings show that the CCL19/CCL21-CCR7 and CXCL13-CXCR5 axes are vital for regulating TLS development (49). Although the knowledge of TLS formation mechanisms is widely researched, the possible mechanisms and factors need to be further explored in future studies. The main molecular and cellular mechanisms of TLS formation are shown in **Figure 1**.

## The Function of TLSs in the TME

### Favorable Impact of TLSs on Antitumor Properties

Mature TLSs are similar to SLOs, which contain T cell-rich areas with CD3^+^ T cells and dendritic cell (DC)-lysosomal associated membrane protein^+^ (DC-LAMP) mature DCs and follicular CD20^+^ B cell-rich zones (12). The B cell follicles in TLSs comprise follicular dendritic cells (FDCs), B cells, plasmablasts, and TFH cells required for GC formation and B cell differentiation (50). Macrophages, neutrophils, and regulatory T (Treg) cells have been discovered in the TLSs of lung cancer, pancreatic cancer, and ovarian cancer (51–54). TLSs are divided into classical and nonclassical structures. Classical structures are mature and contain T cells, DCs, B cells, and FDC compartments and comprise more active components than nonclassical structures, mainly B cells. Nonclassical TLSs usually contain B cells that are less activated than those in classical structures (14). TLSs are distributed intratumorally and peritumorally and are more abundant in the invasive margin than in the tumor core (12). Intratumoral TLSs may have greater prognostic significance, but this has not been widely established. In some studies, intratumoral TLSs are a favorable prognosticator in pancreatic cancer and hepatocellular carcinoma (20, 21). One study proposed a hypothesis to explain the better prognosis of intratumoral TLSs. Tumors with, rather than without, intratumoral TLSs are less invasive, especially regarding blood vessel invasion, and have a role related to the immune response. These tumors retain a relatively complete vascular network to transport immune cells and other molecules into the tumor and initiate a more effective antitumor immune response (17).

**Figure 2:**
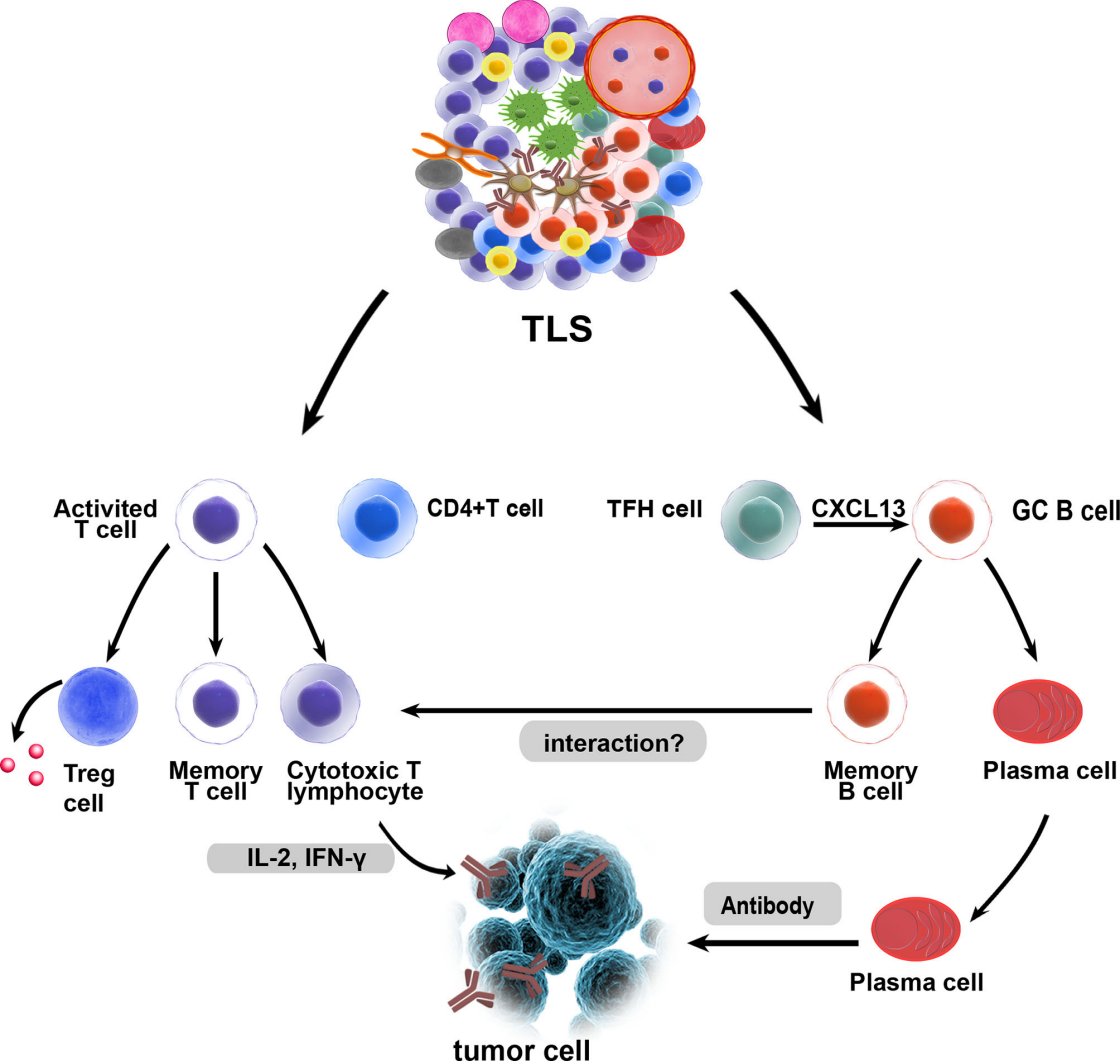
Potential functions of tertiary lymphoid structures in the TME. As in canonical SLOs, TLSs may constitute a critical site where specific T and B cells can undergo terminal differentiation into effector cells. GC B cells can differentiate into memory B cells and plasma cells in TLSs, and fully differentiated B cells and exert their antitumor effects. T cells can differentiate and expand, and they are activated as effector cells that exert cytotoxic effects. B cells and T cells in TLSs may interact with each other and play a synergistic role, which needs to be confirmed by more studies. Treg cells within TLSs could exert a negative influence on the capacity of TLSs to generate effector and memory lymphocytes.

Increasing evidence shows that TLSs play an important role in controlling tumor invasion. Mature TLSs exhibit evidence for the formation of GCs (24) and GC B cells in TLSs are characterized by FDCs and Ki67^+^ proliferating B cells (51). Oligoclonal B cell responses have been identified in melanoma, which suggests an active humoral antitumor response within TLSs that is driven by B cells (55). High PC counts are associated with higher numbers of TLSs and B cells in breast cancer and neck carcinomas (56, 57). PCs surrounded by TLSs are associated with the highest levels of TILs and cytotoxicity-related gene products in ovarian cancer. This study showed that CD8^+^ TILs can predict prognosis only in combination with PCs, CD20^+^ TILs, and CD4^+^ TILs, suggesting that these four lymphocyte subsets work in concert to promote antitumor immunity, which indicates that TLSs may facilitate coordinated antitumor responses involving the combined actions of cytolytic T cells and PCs (58). B cells in TLSs are organized and highly differentiated and can produce tumor-specific antibodies in adenocarcinomas and ovarian cancer (59). In omental metastases from ovarian cancer, memory B lymphocytes essentially located within TLSs had higher clonality and somatic hypermutation rates, and they produced chemokines attracting DCs, T cells, and natural killer (NK) cells. The density of B cells also correlated with that of mature DCs in the stroma of tumors (53). Recent studies have shown the important role of TLSs and B cells in immunotherapy. The frequencies of memory B cells, PCs, and GC-like B cells in the tumors of responders treated with immune checkpoint blockade (ICB) therapy are significantly higher than those in nonresponders. Increased B cell proliferation indicating GC activity and formation within TLSs has been observed (60). High expression of B-lineage markers is associated with improved prognosis and TLS formation in sarcoma (61). B cells within TLSs can predict favorable prognosis in melanoma patients receiving ICB therapy. In addition, B cell-rich tumors are associated with elevated levels of initial and memory T cells. T cells in tumors without TLSs possess a dysfunctional molecular phenotype, which indicates that TLSs have a key role in the melanoma TME by conferring distinct T cell phenotypes (62). In summary, these studies demonstrate a major role of TLS-associated B cells in TLS function. B cells probably act together with key immune constituents of TLSs by altering T cell activation and function. Memory B cells may act as antigen-presenting cells (APCs) to drive the expansion of both memory and naive T cell responses. B cells can also activate and recruit other immune effector cells by secreting an array of cytokines (60). The potential functions of TLSs in the TME are shown in **Figure 2**.

The existence of TLSs at metastatic tumor sites is the key factor in the level of TILs, which directly determines the antitumor effect (19). Moreover, the presence of TLSs led to increased infiltration and activation of T cells and other immune cells and was associated with a good prognosis in liver cancer and pancreatic carcinoma (21, 63, 64). There is evidence that TLSs can activate effector T cells in tumors (65). In MC38 tumors, T cells from TLSs exhibited a largely enhanced baseline level of IFN-γ (interferon-gamma) release. This finding revealed successful antitumor T cell priming activity within induced TLSs, and TLSs may act as immune factories where T cells activate effector cells to mediate synergistic antitumor effects (66). Studies of lung and ovarian cancers showed that TLS-associated DCs establish unique immune states characterized by a strong T helper (Th) 1 orientation and facilitate a good prognosis, indicating that antigen presentation allows local T cells to initiate responses to tumor-associated antigens in TLSs (67, 68). Whether TLS-associated DCs present tumor antigens directly to CD8^+^ T cells or whether CD4^+^ Th cells participate in the production of CD8^+^ cytotoxic T cell responses in TLSs remains to be further studied. TFH cells produce CXCL13, potentially resulting in the formation of TLSs to trigger the GC B cell response (69). FDCs can also produce chemokines and cytokines involved in B cell proliferation and migration in LN, such as interleukin (IL) -6 and CXCL13 (70). B cells produce LTα1β2, which has a crucial function in the differentiation of FDCs within TLSs (71).

HEVs in TLSs are associated with T and B cell infiltration and indicate favorable outcomes in oral carcinoma and breast cancer (72, 73). The emergence of HEVs also contributes to the formation of TLSs (74). There is ample evidence that the function of HEVs in TLSs is similar to that in LNs, providing a channel for immune cells to accumulate in the tumor. HEVs in TLSs express molecules similar to those expressed in LNs, such as CCL21 and peripheral node addressing (PNAd), and cells expressing CCR7 and L-selectin ligands for receptors on HEVs are found in TLSs (75). PNAd expression indicates that HEVs are essential for recruiting lymphocytes to lymphoid organs (76), an event that orchestrates the extravasation of Lselectin^+^ and CCR7^+^ immune cells into TLSs (77, 78). LTαβ plays a key role in PNAd expression in HEV (79). Single-cell analysis revealed the heterogeneity of HEVs in LN. LTβR signaling and inflammation also have crucial effects on HEV transcriptomes (80). However, the study showed that HEV neogenesis is dependent on tumor necrosis factor receptor (TNFR) rather than LTβR signaling in Treg-depleted tumors, suggesting another mechanism for HEV formation. The expression of PNAd is not dependent on the LTβR signal but is stimulated by activation of TNFR mediated by LTα3 derived from CD8^+^ T cells (81). HEV formation is associated with increased T and B lymphocyte infiltration and activation in murine pancreatic cancer and breast cancer (82, 83). In mouse models of melanoma and lung cancer, the LN-like vasculature in tumors, characterized by the expression of PNAd and chemokine CCL21, induced by effector lymphocytes allows naive T cells to enter tumors and enhance antitumor immunity. Vasculogenesis is regulated by a mechanism involving CD8^+^ T cells that secrete IFN-γ and LTα3 (84). In summary, T cells may contribute to the formation of the peripheral vasculature and HEV.

### The Adverse Impact of TLSs on Tumors

Nevertheless, a few studies have indicated that TLSs have a negative impact on prognosis in colorectal cancer and breast cancer (25–27). The studies showed that TLSs that develop in the inflamed liver during hepatitis can function as a niche for tumor progenitor cells in hepatocellular carcinoma and are associated with an increased risk of late recurrence and decreased survival. It can be postulated that TLSs, which persist in the liver and are associated with a viral infection, play a different role than TLSs induced by tumors (25). Lymphoid aggregates are associated with more advanced diseases and indicate an adverse prognosis in colorectal cancers. These structures form in association with more advanced tumors, suggesting that they are a reaction to progressive tumor invasion, and their prognostic significance varies with disease progression and according to the inherent immunogenicity of the tumor (26). TLSs are associated with adverse prognosis in renal carcinoma with lung metastasis. TLSs are rarely found in lung metastasis of renal carcinoma, and studies speculated that the presence of T cells may not be educated in peritumoral TLSs but may reflect a chronic inflammatory response, which is known to be harmful to the host. At the same time, the high expression of vascular endothelial growth factor (VEGF) and IL-6 genes in renal carcinoma may also inhibit the differentiation of DCs, resulting in an impaired T cell response and poor prognosis (85). TLS Treg cells are detected in breast and colorectal cancers (86, 87). The decrease in the number of TLS Treg cells is associated with tumor regression in metastatic prostate cancer (88). Treg cells are present in TLSs in tumor-bearing lungs and exhibit activated phenotypes. Costimulatory ligand expression by DCs and T cell proliferation rates increased in TLSs after Treg cell depletion, enhancing the antitumor immune response. The reason may be that Treg cells in TLSs regulate DC function by reducing costimulatory levels, the immunosuppression of Treg cells to DCs is relieved after Treg cell depletion, and the TLS microenvironment may become more immunostimulatory to promote antitumor responses by T cells (54). The recruitment of Treg cells and myeloid-derived suppressor cells (MDSCs) to lymphoid aggregates in mouse B16 melanomas expressing CCL21 was found to correlate with the promotion of tumor growth (89). Therefore, TLS-associated Treg cells and MDSC presence may exert a negative influence on the capacity of TLSs to generate effector and memory lymphocytes.

## Development of Multiple Approaches to Induce the TLS Formation

A variety of LN modifications to improve the efficacy of tumor immunotherapy have been widely discussed and researched. Targeting LN can affect the efficacy of cancer vaccines, ICB therapy, and adoptive cell transfer (ACT) at the cellular level. Macroscopic biomaterials mimicking LN characteristics can be used as immune niches for cell reprogramming and *in vivo* transmission and can be used for preclinical testing of drugs and vaccines *in vitro* at the tissue level (90). TLSs may be the first line of T cell differentiation and expansion and are the key to inducing intratumoral immune sensitization in situ. Therefore, similar principles can be used for developing strategies to induce TLS formation, and a new antitumor immune strategy can be constructed. Although biomaterials for transporting or recruiting APCs can mimic the cellular characteristics of SLOs, other strategies aim to induce TLS formation specifically, as observed *in situ*. These strategies aim to mimic the chemokine and inflammatory signals of the main molecular and cellular mechanisms of TLS formation. In the next section, we discuss strategies that induce TLS formation through the delivery of chemokine-expressing cells or chemokines, implantation of biomaterial scaffolds containing these inflammatory factors and agents, and multiple therapeutic approaches.

### Chemokines and Cytokines

A chemokine delivery strategy for TLSs provides a convenient way to generate ectopic lymphoid tissue in tumors. Recent electronic screening techniques involving the identification of TLS-related chemokine genes that induce lymphocyte chemotaxis have offered a framework for a more effective design of TLSs (91, 92). A 12-chemokine gene signature also provided a promising starting point for the potential construction of designed TLSs (93–96). In early studies, chemokines produced by lymphoid structures were expressed in various ways, which led to the formation of lymphoid tissue structures. For example, transgenic mice expressed B lymphocyte chemokines in pancreatic islets, and the expression of B lymphocyte chemokines resulted in the formation of LN-like structures that included HEVs, interstitial cells, and B and T cell zones and illustrated that the maintenance of B lymphocyte chemokine-induced lymphoid structures depends on LTβR signaling (97). CCL21 exhibits a stronger capacity than CCL19 to induce more organized infiltrates in the islets of transgenic mice (98). Intratumoral injection of CCL21 facilitated lymphocyte infiltration into pancreatic tumors (99), and targeting lymphotoxin-α can induce lymphocyte infiltration and lymphoid-like tissue formation in B16 melanoma (100). LN-like lymphocyte infiltration was also found in transgenic mice expressing CCL21 driven by the thyroglobulin promoter in the thyroid gland and transgenic pancreas (101, 102). Type I interferon can also drive B cell recruitment by CXCR5–CXCL13 signaling and initiate ectopic GC formation within TLSs in pulmonary virus infection (103). In the salivary glands of adult mice, IL-7 regulates lymphatic vessel expansion and promotes the neogenesis of TLSs in the first phase, and LTβR signaling regulates TLS neogenesis in the second phase (104). Th17 cytokines can regulate TLS development and function. For instance, IL-22 modulates CXCL12, CXCL13, and IL-23 production, contributing to the formation of TLSs (105–107). In conclusion, these studies show that many chemokines and cytokines involved in lymphoid structure formation can be used as novel and feasible inducers in combination with other stimulants and multiple methods to induce the formation of TLSs.

**Figure 3:**
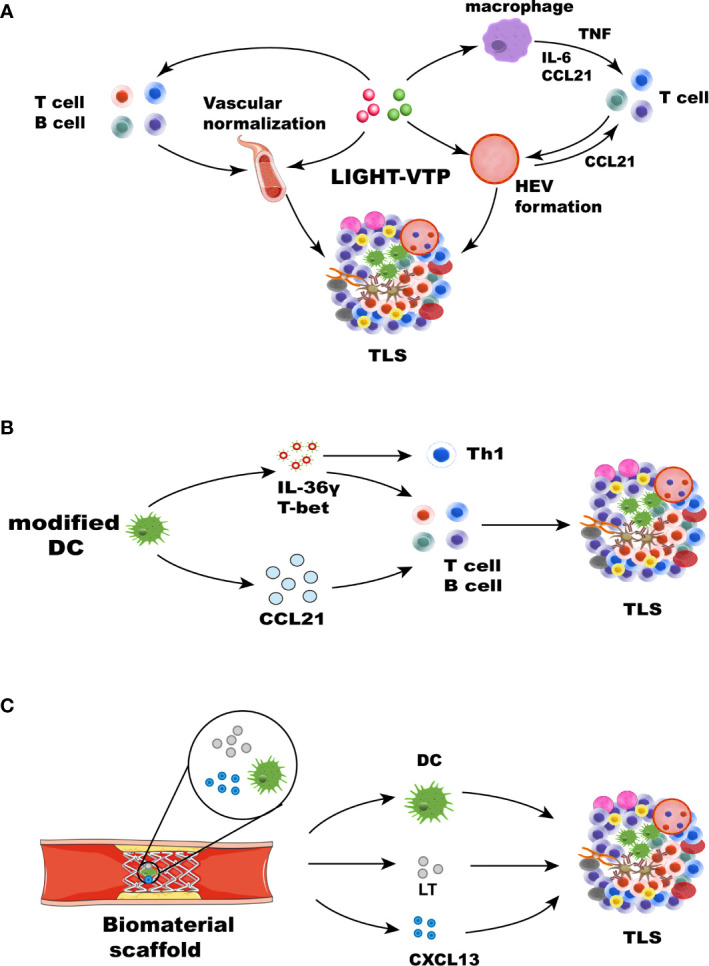
Strategies for therapeutic induction of TLS formation. TLS inducers, such as chemokines, cytokines, DCs, and therapeutic approaches, can induce TLS formation in different ways. **(A)** Cytokines and chemokines involved in lymphogenesis, including LIGHT, CCL21, CXCL13, LT, and IFN-γ, can lead to the formation of TLSs. LIGHT-VTP targeting tumor vessels can induce vessel normalization, and HEV and TLS formation may occur through a self-amplifying loop. The mechanism may be related to the LIGHT-triggered expression of the inflammatory cytokines IL-6, TNF, and CCL21 in macrophages. These chemokines can recruit T cells. Macrophages and T cells play an important role in the formation of HEVs and TLSs (83). HEV formation and vascular normalization can also recruit more immune cells. **(B)** Some immune cells, such as modified DCs and stromal cells, leading to the formation of TLSs. DCs engineered to secrete IL-36γ also initiate therapeutic TLS formation, which can upregulate the expression of T-bet. T-bet and IL-36γ cooperate to reinforce their expression and recruit immune cells, leading to TLS formation. DCs modified with the CCL21 gene can significantly increase T cell infiltration. Activated DCs can also upregulate many factors associated with TLS formation. **(C)** Biomaterials can provide 3D scaffolds in *situ* and deliver cells and chemokines. Collagen scaffolds containing LT, CCL21, CXCL13, and activated DCs were transplanted into mice. The recruited lymphocytes can form artificial lymph node structures.

LIGHT, the 14th member of the tumor necrosis factor superfamily (TNFSF14), is a protein primarily expressed on activated T cells and immature DCs (108). LIGHT can function as both a soluble and cell surface-bound type II membrane protein and interact with its two primary functional receptors: Herpes Virus Entry Mediator and LTβR (109). LIGHT can interact with Herpes Virus Entry Mediator and deliver co-stimulatory signals to T cells (44). LTβR is found on the surface of epithelial, stromal, and myeloid cells (110). LIGHT-LTβR signaling plays an important role in immune responses, functioning to repair tumor vasculature and to support effector cells cell trafficking to and infiltration into tumors (111). Recently, LTβR signal transduction induced by LIGHT has become a focus of the investigation. When combined with an anti-VEGF antibody, LIGHT can activate LTβR signaling and mediate chemokine production to recruit T cells (112). In pancreatic cancer, targeting LIGHT for homing to tumor vessels *via* a vascular targeting peptide (VTP), LIGHT-VTP showed a dual ability to induce TLS formation and regulate the angiogenic vasculature (83). LIGHT targeting to tumor vessels induces vessel normalization, and HEVs and TLS formation may occur through a self-amplifying loop in pancreatic cancer. The mechanism may involve the LIGHT-triggered expression of inflammatory cytokines in macrophages, such as IL-1β, IL-6, CXCL13, TNF, and CCL21, These chemokines further recruit T cells. Macrophages and T cells have been deemed essential for HEV and TLS formation (83, 113) (**Figure 3A**). LIGHT-VTP in combination with ICB therapy can produce intratumoral memory T cells and Teff cells and improve prognosis (114). In addition, LIGHT-VTP combined with anti-VEGF and ICB therapy can increase the frequency of HEVs and normalize tumor vessels and the accumulation of T cells in glioblastoma and lung metastases (115, 116). The LT-LIGHT axis provides key differentiation signals guiding the differentiation of the reticular network and vascular system, maintaining the mesenchymal differentiation pathway of the specialized network, and remodeling reactive LNs (117). The LTβR signaling pathway plays a critical role in HEV differentiation and function in LN (44). Because of the similarity between SLOs and TLSs, it is speculated that LTβR signaling is also involved in HEV differentiation and function in TLSs. Further studies are required to understand the precise mechanisms by which HEV formation in TLSs is induced and the effects of HEVs on different types of cancer. This knowledge may guide the therapeutic objectives of cancer interventions. Other means can also be used to deliver LIGHT to tumor sites, and the oncolytic activity of attenuated *Salmonella Typhimurium* was enhanced by the stable insertion of the gene encoding LIGHT. Attenuated *S. Typhimurium* expressing LIGHT inhibited the growth of primary tumors and the spread of lung metastasis (118). The findings suggest that avirulent bacteria can be used as targeted carriers for the local production of therapeutic proteins in tumors. In recent years, the potential use of exosomes in the treatment and control of many diseases has expanded because of their inherent characteristics in regulating complex intracellular pathways. The characteristics of exosomes can also be exploited to induce TLSs. Exosomes are extracellular vesicles derived from endosomes and have a diameter of approximately 40-160 nm. They can carry a variety of substances, such as proteins and DNA, to allow these substances to be absorbed by other cells (119, 120). Therefore, we hypothesize that exosomes can be used as carriers to load many chemokines and cytokines to induce the formation of TLSs.

Toll-like receptors (TLRs) have also been researched concerning TLS formation. Myofibroblasts were stimulated with TLR agonists and cytokines in giant cell arteritis, which upregulated B cell-activating factor and CXCL13 and resulted in the formation of TLSs (121). Inhalation of TLR9 agonists can generate profound remodeling of tumor-bearing lungs and lead to TLS formation in adjacent tumors (122). In addition, both the anti-HBV response to the TLR7 agonist GS-9620 and TLR4 agonists in mouse models of myasthenia gravis can induce TLS generation (123, 124). Transforming growth factor-β (TGF-β) plays a noncanonical role in coordinating immune responses against ovarian cancer. CD8^+^ T cells in the presence of TGF-β upregulate the secretion of CD103 and CXCL13, and CD8^+^ TILs play a role in mediating B cell recruitment and TLS formation (125).

### Cells

An alternative approach to produce TLSs is to deliver cells that express chemokines or to engineer chemokines that are associated with lymphomagenesis. DC-based therapeutic strategies can be used therapeutically to promote the extranodal priming of antitumor immunity (126). DCs expressing T cell chemokines were injected into melanoma tumors, which yielded rapid T cell infiltration and initiation of intratumor responses (127). Additionally, intraperitoneal injection of murine DCs promoted the acute infiltration of immature T cells and NK cells into the TME, an effect related to upregulated expression of NK and T cell recruitment chemokines by murine DCs (128). DCs engineered to overexpress T-bet suppressed the growth of sarcomas *in vivo* after intratumoral injection and prolonged the overall survival of mice (126, 128). DCs promote LT signaling through LTβR for HEV differentiation and function in LN (129). DCs, which coordinate adaptive immune responses, have historically been a promising target. DCs are a source of LT, and homeostatic chemokines (CXCL12, CXCL13, CCL19, and CCL21) are known to contribute to TLS formation in the lungs of influenza virus-infected mice. Similar to the depletion of DCs, blockade of LTβR signaling after virus clearance leads to the disintegration of TLSs and GC reactions. It is suggested that the DC-mediated LTBR pathway contributes to the formation of TLSs (36). Other methods have focused on modifying and editing DCs to express the transcription factor T-bet and secrete IL-36γ to play a vital role. IL-36 cytokines are an IL-1 subfamily consisting of three agonists that signal through the common heterodimeric receptor IL-36R (130), which is expressed on endothelial cells and many immune cells, including T cells and DCs (131). IL-36γ is involved in polarizing type-1 immune responses. It is a downstream target of the type-1 transactivator T-bet and can induce T-bet expression in target cells (132). Research has shown that IL-36γ predominantly expressed by M1 macrophages and vasculature cells, including smooth muscle cells and HEVs, mediates polarization toward a type 1 immune response. This pattern of IL-36γ expression increased CD4^+^ central memory T cell infiltrate and the density of B cells and led to TLS formation in human colorectal cancer (133). The injection of tumors with DCs engineered to secrete a bioactive form of mIL-36γ also initiated therapeutic TLS formation and slowed tumor progression in a mouse model of colorectal carcinoma. Furthermore, DC.IL-36γ cells show strongly upregulated expression of T-bet, suggesting that T-bet and IL-36γ cooperate to reinforce each other’s expression in DCs, rendering them competent to promote TLS formation in the therapeutic TME (134) (**Figure 3B**). In lung cancer, autologous DCs transduced with an adenoviral vector modified with the CCL21 gene significantly reduced the tumor load and T cell infiltration (135), accompanied by enhanced expression of IFN-γ, IL-12, and CXCL10, as well as molecules related to reduced immunosuppression in the TME (136). Mice vaccinated with DCs charged with apoptotic/necrotic B16 cells are protected against B16 challenge, and TLSs form at the vaccination site (137). In conclusion, DCs play an important role in inducing TLS formation. These results provide a framework for the usage of DCs. Promoting the expression of multiple chemokines by targeting DCs is a valuable strategy to induce TLS formation.

LTi-like cells from newborn mouse LNs were injected intradermally into adult mice and formed TLSs in the skin, and the results indicated that hyperactivated lymphocytes can fulfill the role of LTi cells during inflammatory responses (138). Subcutaneous injection of the LN-derived stromal cell line resulted in the formation of TLSs that promote infiltration of immune cell subsets and inhibit tumor growth by improving the antitumor activity of TILs (66). Lymphoid tissue-like organoids were constructed by transplantation of stromal cells embedded in biocompatible scaffolds into the renal subcapsular space in mice. The structure is similar to that of SLOs and contains clusters of B and T cells and HEVs, DCs, and FDC networks (139). Other cells, such as immune fibroblasts, bone marrow mesenchymal stem cells (MSCs), adipocytes, and macrophages, also play their roles. Research on autoimmune conditions demonstrated that external triggers at mucosal sites can induce gradual differentiation of stromal cell populations into immune fibroblast networks, which supports the establishment of TLSs at an early stage. This process is mediated mainly by paracrine and autocrine signals regulated by IL-13. Once lymphocytes are recruited, the initial fibroblast network is expanded by local production of IL-22 and lymphotoxin. This finding demonstrates the role of immune fibroblasts in maintaining TLS and supporting their formation and identifies new therapeutic targets (140). Human MSCs stimulated with TNF-α and IL-1β significantly increased the expression of CCL19, VCAM1, ICAM1. Stimulated MSCs can induce CD4^+^ T cell proliferation. MSCs could play a key role as LTo cells in promoting the early inflammatory and initiating the formation of kidney-specific TLSs (141). Mucosa**-**resident CXCL13^+^CX3CR1^hi^ macrophages are responsible for recruiting B cells and CD4^+^ T cells to sites of *Salmonella* invasion and subsequently activating them, resulting in TLS formation and a local pathogen-specific IgA response (142). Recently, the combination of TNF-α and lipopolysaccharide was shown to directly induce adipocytes to produce TLS-related chemokines, thereby coordinating the formation of functional TLSs in the mesentery affected by Crohn’s disease (143). In summary, these studies have further proved the various initiating factors and mechanisms of the formation of TLSs and provided more references and insights for inducing the TLS formation.

### Biomaterials

Biomaterials can support the formation of TLSs by locally and controllably releasing chemokines and providing cellular support. Scaffolds are usually three-dimensional microporous structures designed to achieve cell encapsulation *in vitro* or cell penetration *in vivo* while providing mechanical support, cell adhesion capability, and a continuous supply of biological cues to promote cell migration and interaction (144). Biomaterial scaffolds can boost the efficacy of immunotherapies, such as cancer vaccines and ACT (145–147). For instance, biomaterials loaded with signaling molecules and engineered T cells have been evaluated *in vitro*. These biomaterials were surgically implanted near the tumor or under a resected tumor bed, where they maintained continuous proliferation and release of specific T cells (148).

In early cases, collagen scaffolds containing both thymus-derived stromal cells expressing LTα and activated DCs were transplanted into the renal subcapsular space of mice. The recruited lymphocytes formed artificial lymph nodes (ALN) structures, which contained FDC, T cell, and B cell regions and HEV-like structures. ALN induced a potent immune response *in vivo* and the accumulation of memory and effector T and B cells. The engineered structures elicited a humoral response after vaccination and could be transplanted into immunodeficient mice to secrete antibodies after secondary immunization (139, 149, 150) (**Figure 3C**). Based on this strategy, cell-free biomaterials have been explored. Hydrogels can provide a controlled cellular microenvironment for immune cells so that they can be recruited, expanded, and activated *in vitro* and *in vivo* (151). Hydrogels can be used to deliver antigens, chemokines, and other factors to DCs and induce T and B cell responses, and they can effectively encapsulate immunomodulators and immune cells. DCs can be activated *in vitro* in hydrogels before implantation and can be recruited and activated in gels by immobilized stimulators, as in a bioreactor (152, 153). In another study, collagen sponge scaffolds embedded with sustained-release gel beads containing LTα1β2, and many chemokines were transplanted into the subcapsular space of mice to establish ALN-like TLSs, recruiting memory T cells and B cells and induced a strong antigen-specific immune response (154). A synthetic immune priming center consisting of an *in situ* cross-linking hydrogel delivering chemokines and particles loaded with DNA and siRNA can attract numerous DCs and can both generate a strong transition to a T helper 1 response and increase the cytotoxic T lymphocyte (CTL) response. The multimode injectable system can simultaneously deliver chemokines, DNA, and siRNA antigens to DCs. This system constitutes a novel strategy to regulate immunotherapy *in situ* and could provide an effective vaccine strategy to prevent cancer (155).

Other biomaterials include polylactide-coglycolide (PLG), nano-sapper, and nanoparticles. A macroporous PLG matrix was used to deliver granulocyte-macrophage colony-stimulating factor (GM-CSF), tumor antigens, and danger signals *in vivo*. GM-CSF recruited DCs and significantly enhanced their homing to LNs, and danger signals and cancer antigens further activated the recruited DCs. These materials elicited protective antitumor immunity and showed prospects as cancer vaccines (156). A study demonstrated improved immune function by targeting DCs with adjuvant vector cells engineered from MKT cell ligands loaded with tumor antigen mRNAs. This method also enhanced the local immune response *via* TLS formation (157). Importantly, these polymers may be designed to program the transport of various types of cells *in vivo*. Nano-sapper was co-loaded with an antifibrotic protein and a plasmid expressing LIGHT. By normalizing the tumor vasculature, reducing collagen deposition, and stimulating the expression of lymphocyte-recruiting chemokines, Nano-sapper induces TLS formation to promote CTL infiltration and remodel the TME (158). Recognition of ectopic HEVs in human pancreatic ductal adenocarcinoma by engineered MECA79-coated nanoparticles can increase the transport of Taxol to the tumor and distinctly reduce tumor growth (159). Nanomaterials are promising for inducing TLS formation. Local delivery of engineered biomaterials can play a role by establishing synthetic immune niches to enhance antitumor immunity. Immunotherapy based on biomaterials will facilitate the development of the next generation of tumor therapies.

### Other Therapeutic Approaches

Multiple cancer therapeutic strategies, such as cancer vaccines, ICB therapy, antiangiogenic therapy, radiotherapy, and chemotherapy, contribute to TLS formation. After therapeutic vaccination against human papillomavirus serotype 16 with E6/E7 antigens, significant immune changes in the TME were observed in subjects with CIN2/3, and TLSs formed in the immune-infiltrated cervical tissues. At the molecular level, these histological changes in the matrix were characterized by increased gene expression and associated with immune activation (CXCR3) and effector function (T-bet and IFN-β) (160). A prominent study of patients with resected pancreatic cancer showed that 33 of the 39 patients treated with the GM-CSF vaccine exhibited TLS formation after 2 weeks. Further analysis showed that these structures could regulate adaptive immunity. Inhibition of the Treg signaling pathway and enhancement of the Th17 signaling pathway in TLS aggregates were associated with increased survival and intratumoral Teff: Treg ratios and upregulation of the mechanism of immunosuppression (161). The findings help to guide the production of the next generation of effective cancer vaccines and facilitate better responses to ICB therapy. TLSs containing lymphocytes and APCs appeared in all 11 patients who received cisplatin neoadjuvant chemotherapy in a study on hepatoblastoma, indicating that cisplatin can induce TLS infiltration and synergistically induce the death of immunogenic cells and trigger an antitumor immune response. This may involve so-called immunogenic cell death, a controlled cell death process that produces damage-associated molecular patterns that can be used as adjuvants to initiate an immune response through the recruitment and activation of DCs (162). Administration of preoperative chemoradiotherapy (neoadjuvant chemotherapy, NAC) was associated with increased TLS formation and may affect the immunological composition of the TME and confer a favorable prognosis in patients with pancreatic ductal adenocarcinoma (163). However, corticosteroid therapy during chemotherapy impaired GC formation and reduced TLS prognostic value in patients with lung cancer (164). After radiotherapy, apoptosis in tumors with TLSs increased significantly. The TLSs also showed an acute increase in apoptosis and size reduction. Although their size tended to normalize after 2 weeks, the apoptotic rate remained high, suggesting active and continuous proliferation in residual irradiated cells and providing them with a window to optimize their unique function (165). Low-dose stimulator of interferon genes (STING) agonist treatment can upregulate the expression of various cytokines and increase the infiltration of T cells and DCs to establish a proinflammatory TME, which can also lead to normalization of the tumor vasculature, ultimately inducing the formation of TLSs and controlling tumor growth. Stimulating DC maturation and local production of vascular normalization-promoting and TLS-promoting factors, such as CCL19, CCL21, LTα, LTβ, and LIGHT (166). A study showed that Treg elimination can activate CD8^+^ T cells and promote the development of HEVs in tumors. The study proposed a model in which a positive feedback loop of T cell activation by Treg cell depletion can promote HEV development, T cell infiltration, and tumor destruction (81). A prostate cancer study showed that Treg cells and cyclooxygenase 2 are attractive therapeutic targets that can be used to strengthen TLS-driven tumor immunity. In particular, the existence of HEVs and lymphatic vessels suggests that TLSs can also be used as a platform for cell-based or cyclooxygenase 2 blockade therapy to control tumor growth (88).

PD-L1^+^, PD-L2^+^, LAG3^+^, and TIM3^+^ cells were detected in some breast cancer-related TLSs, and PD-1 was used as a marker of T cell activity in both the T and B cell areas in TLSs. The expression levels of immune checkpoint molecules were associated with the level of TILs and TLS formation (167). In a group of patients with renal carcinoma, the low expression of immune checkpoints and the localization of mature DCs in TLSs are associated with a better prognosis (168) (NCT03387761). Recently, some prominent studies have shown that B cell and TLS formation promote the immunotherapy response in patients with melanoma and sarcoma after ICB therapy (60–62). In a study of locoregionally advanced urothelial carcinoma, the formation of TLSs was observed in responding patients after treatment with combined CTLA-4 and PD-1 blockade therapy, which could be an effective preoperative treatment strategy (169). Another study compared the metabolic, transcriptional, and functional characteristics of intratumoral CD8^+^ T cell subtypes with high, moderate, and no PD-1 expression from patients with non-small cell lung carcinoma. PD-1^+^ high T lymphocytes produce CXCL13, which mediates the recruitment of immune cells to TLSs and has the potential to be predicted after treatment with PD-1 blockade therapy (170). Combination therapy with anti-VEGFR2 and anti-PD-L1 antibodies can induce HEV formation in pancreatic and breast cancer. LTβR signaling plays an important role in the generation and activation of tumor HEVs. HEV formation can increase the activity of CTLs, which makes tumors sensitive to ICB therapy (82). An anti-mouse LTβR agonistic antibody increased TIL infiltration in a mouse model of colon cancer. Agonistic monoclonal antibodies targeting LTβR are a novel method for treating colorectal cancer and potentially other types of cancer (171). Considering that the formation of TLSs is strongly related to the LTBR signaling pathway, targeting LTβR can also be used as an approach to induce TLS formation and enhance antitumor immunity.

TLSs formation induced by multiple therapeutic strategies may involve a complex network of mechanisms, such as various types of cells, chemokines, and molecular mechanisms. We speculate that the main reason for TLS formation may be that the immune suppression in the TME is relieved after multiple therapeutic strategies, and the function of many immune cells can be restored. These cells interact with each other to activate LTβR signaling and other pathways and induce the production of various chemokines and cytokines, which can ultimately lead to the formation of TLSs. TLSs formation further enhances antitumor immunity, which may explain why the existence of TLSs is related to a more favorable prognosis after therapy.

## Conclusion and Future Perspectives

In summary, current research has revealed the significance of TLSs in tumor immunotherapy. TLSs may constitute a privileged niche for educating T cells and B cells, which can activate and enhance immune responses. Although the major cellular and molecular mechanisms of TLSs have been elucidated, how to utilize them as an important part of the immune-related cancer control strategy is still being developed. Targeting the molecular pathways of TLSs development to induce formation is a promising immunotherapeutic strategy, which may directly enhance the antitumor response in situ. HEV induction therapy deserves more research in the design of new immunotherapies, and a more in-depth understanding of the mechanisms in terms of the types of cytokines and chemokines leading to the formation of HEVs in different types of cancer is needed. In the future, we need to focus on the combination of methods inducing HEV and TLSs formation with new therapeutic strategies that can alleviate immunosuppression, such as chemotherapies, radiotherapies, and ICB therapies. These strategies may promote the formation of TLSs as well to synergistically enhance adaptive immunity and provide insight into ultimately effective immune-mediated tumor control.

## Author Contributions

Conceptualization: WK and ZH. Writing original draft: WK. Supervision: ZF and PR. All authors contributed to the article and approved the submitted version.

## Funding

This work was supported by funding from the National Natural Science Foundation of China [grant numbers: 82071986, 81771827] and the Province Natural Science Foundation of Hunan [grant numbers: 2020JJ4855, 2020JJ4841].

## Conflict of Interest

The authors declare that the research was conducted in the absence of any commercial or financial relationships that could be construed as a potential conflict of interest.

## Publisher’s Note

All claims expressed in this article are solely those of the authors and do not necessarily represent those of their affiliated organizations, or those of the publisher, the editors and the reviewers. Any product that may be evaluated in this article, or claim that may be made by its manufacturer, is not guaranteed or endorsed by the publisher.

## Abbreviations

TLSs, tertiary lymphoid structures; TILs, tumor-infiltrating lymphocytes; TME, tumor microenvironment; PD-1, programmed cell death protein-1; PD-L1, programmed death-ligand 1; SLOs, secondary lymphoid organs; GCs, germinal centers; HEVs, high endothelial venules; LN, lymph node; DCs, dendritic cells; Teff, effector T cell; FDCs, follicular DCs; PCs, plasma cells; PNAd, peripheral node addressing; TFH cells, T follicular helper cells; Treg cells, regulatory T cells; LTi, lymphoid tissue inducer; LT, lymphotoxin; LTβR, lymphotoxin-β receptor; IL, interleukin; ICB, immune checkpoint blockade; APCs, antigen-presenting cells; TGF-β, transforming growth factor-β; TNFR, tumor necrosis factor receptor; TNF-α, tumor necrosis factor-α; ACT, adoptive cell transfer; VEGF, vascular endothelial growth factor; VTP, vascular targeting peptide; TLRs, Toll-like receptors; IFN-γ, interferon-gamma; ILC3s, group 3 innate lymphoid cells; CTL, cytotoxic T lymphocyte; PLG, poly (lactide-coglycolide); TRAIL, TNF-related apoptosis-inducing ligand; MDSCs, myeloid-derived suppressor cells; GM-CSF, granulocyte-macrophage colony-stimulating factor; ICAM-1, intercellular cell adhesion molecule 1; VCAM1, vascular cell adhesion molecule 1; MADCAM1, mucosal addressable cell adhesion molecule 1.
